# A novel peptide with potent and broad-spectrum antiviral activities against multiple respiratory viruses

**DOI:** 10.1038/srep22008

**Published:** 2016-02-25

**Authors:** Hanjun Zhao, Jie Zhou, Ke Zhang, Hin Chu, Dabin Liu, Vincent Kwok-Man Poon, Chris Chung-Sing Chan, Ho-Chuen Leung, Ng Fai, Yong-Ping Lin, Anna Jin-Xia Zhang, Dong-Yan Jin, Kwok-Yung Yuen, Bo-Jian Zheng

**Affiliations:** 1 Department of Microbiology, The University of Hong Kong, Hong Kong, China; 2 School of Biomedical Sciences, The University of Hong Kong, Hong Kong, China

## Abstract

A safe, potent and broad-spectrum antiviral is urgently needed to combat emerging respiratory viruses. In light of the broad antiviral activity of β-defensins, we tested the antiviral activity of 11 peptides derived from mouse β-defensin-4 and found that a short peptide, P9, exhibited potent and broad-spectrum antiviral effects against multiple respiratory viruses *in vitro* and *in vivo*, including influenza A virus H1N1, H3N2, H5N1, H7N7, H7N9, SARS-CoV and MERS-CoV. The antiviral activity of P9 was attributed to its high-affinity binding to viral glycoproteins, as well as the abundance of basic amino acids in its composition. After binding viral particles through viral surface glycoproteins, P9 entered into cells together with the viruses via endocytosis and prevented endosomal acidification, which blocked membrane fusion and subsequent viral RNA release. This study has paved the avenue for developing new prophylactic and therapeutic agents with broad-spectrum antiviral activities.

Pandemics and epidemics of respiratory viruses have been an important source of mortality and morbidity among the human populations. The recurrent outbreaks of highly pathogenic H5N1 influenza A virus infection in poultry with sporadic transmission to human since 1997 resulted in high mortality rate of ~50%^1^,^2^. The intermittent outbreaks of H7N9 influenza since 2013 killed ~40% of the inflicted individuals^2^,^3^. In addition, the emergence of severe acute respiratory syndrome (SARS) in 2003^4^ and Middle-East respiratory syndrome (MERS) in 2012^5^, which resulted in ~10% and ~40% mortality rates, respectively, have raised concerns on the potential outbreaks of fatal viruses that are previously unknown to the human community. In view of the limited efficacy of current vaccines and antiviral drugs against these respiratory viral infections^4^,^5^,^6^,^7^ and the emergence of new respiratory viruses, new agents with broad-spectrum antiviral effects are in desperate need for the prevention and treatment of respiratory viral infections.

β-defensins are a family of endogenous cysteine-rich peptides, which typically contain a six-cysteine motif linked by three intramolecular disulfide bonds at the 1–5, 2–4 and 3–6 positions^8^. β-defensins are secreted from epithelial cells of the mucous membrane on the respiratory, digestive, urinary and reproductive tracts to provide a first-line defense against various infectious pathogens, including a number of enveloped viruses^9^,^10^,^11^,^12^,^13^. The antiviral activity of β-defensins is thought to be mediated through either indirect interaction or direct interaction with viral glycoproteins and/or envelopes^14^,^15^. For example, β-defensins could indirectly interact with viral receptors and/or glycoproteins through the target cells to inhibit virus-receptor binding or virus-cell membrane fusion or to down-regulate cell-signaling pathways required for viral replication^15^,^16^. β-defensins might also directly interact with viral glycoproteins and/or disrupt viral envelopes to inhibit viral entry into the target cells^17^,^18^. Recently, we also found that mouse β-defensin-4 (mBD4) has strong *in vitro* and *in vivo* antiviral effects against H5N1 and H1N1 influenza viruses and SARS-CoV (unpublished data).

Despite the remarkable antiviral activities against different enveloped viruses, the development of defensins as therapeutics has been hindered by several factors, such as suboptimal efficacy, side effects and the lack of cost-effective means of large-scale production^19^. Short peptides derived from human β-defensin-3 have recently been shown to contain more potent antibacterial effects and lower toxicity to host cells than those of human β-defensin-3^20^. Therefore, we designed and synthesized short peptides derived from mBD4 for further investigation. In this study, we identified that a 30-amino-acid short peptide derived from mBD4, P9, could inhibit a broad range of respiratory viruses *in vitro* and *in vivo*. In addition, with a series of experiments, we unveiled the unique antiviral mechanism of P9.

## Results

### The short peptide P9 exhibited the highest antiviral activity among mBD4-dervied peptides, smBD4, and rmBD4

**Figure 1:**
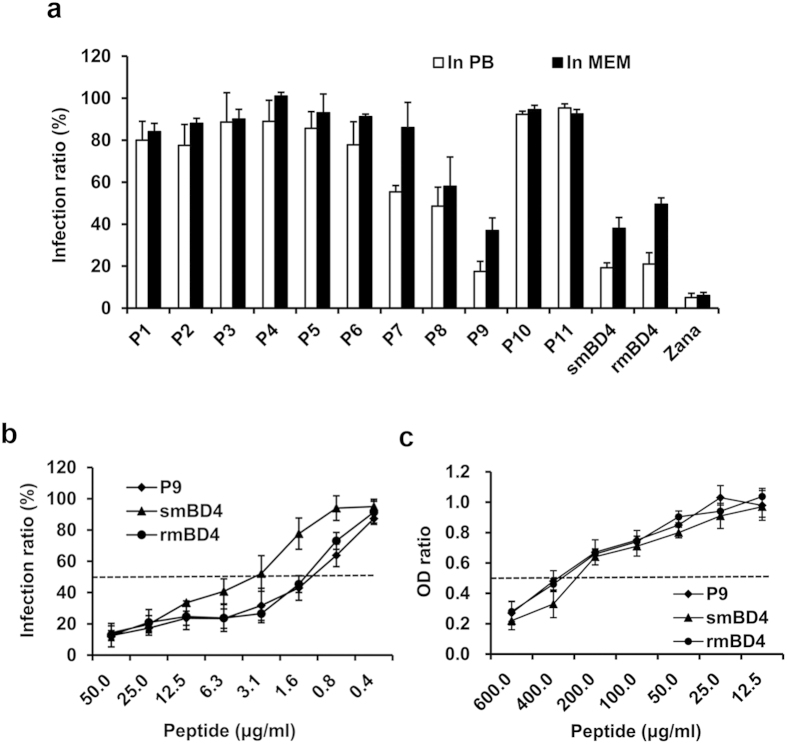
Evaluation of antiviral activity and cytotoxicity of peptides *in vitro*. (**a**) Antiviral activities of smBD4, rmBD4 and 11 short peptides (25 μg/ml) against H1N1 viral infection were tested in MDCK cell cultures. White and black colors indicate that the virus was pretreated with peptides in PB and MEM, respectively. Zanamivir (Zana) was included in the experiment as positive control. (**b**) Anti-H1N1 efficacies of P9, smBD4 and rmBD4 at different concentrations were detected in cultured MDCK cells. The inhibitory activity was directly determined by plaque assay and the infection ratio was calculated as plaque numbers in treated samples/plaque numbers in untreated samples. The IC_50_s are indicated by dotted line, indicating that the mean IC_50_s of P9, smBD4 and rmBD4 are 1.2 μg/ml, 3.2 μg/ml and 1.5 μg/ml, respectively. (**c**) The cytotoxicity of P9, smBD4 and rmBD4 was determined by MTT assay. TC_50_s are indicated by dotted line. All data are presented as means ± SD from three independent experiments.

Eleven mBD4-derived peptides were designed and synthesized (Table 1). Their antiviral activity against influenza virus H1N1 was evaluated in MDCK cells. Among all short peptides, P9 showed the strongest antiviral activity (Fig. 1a). Interestingly, the dose-dependent antiviral effect of P9 was stronger than those of synthetic mBD4 (smBD4) and recombinant mBD4 (rmBD4). In particular, the 50% inhibitory concentration (IC_50_) of P9 was 1.2 μg/ml, which was lower than those of smBD4 (3.2 μg/ml) and rmBD4 (1.5 μg/ml) (Fig. 1b). Furthermore, the 50% toxic concentrations (TC_50_) of P9, smBD4 and rmBD4 were 380, 280 and 360 μg/ml, respectively (Fig. 1c). The selectivity index of P9 was 316, which was higher than those of smBD4 (87) and rmBD4 (240). Notably, both of the liquid form and powder form of P9 demonstrated stable shelf lives as they exhibited similar antiviral activity after storing at −20 °C for more than 1 year (Supplementary Fig. 1). Taken together, among smBD4, rmBD4 and the 11 peptides derived from mBD4, P9 exhibited the highest antiviral activity and selectivity index. Thus, P9 was selected for further studies.

### P9 protected mice against lethal challenge of H1N1 virus

**Figure 2:**
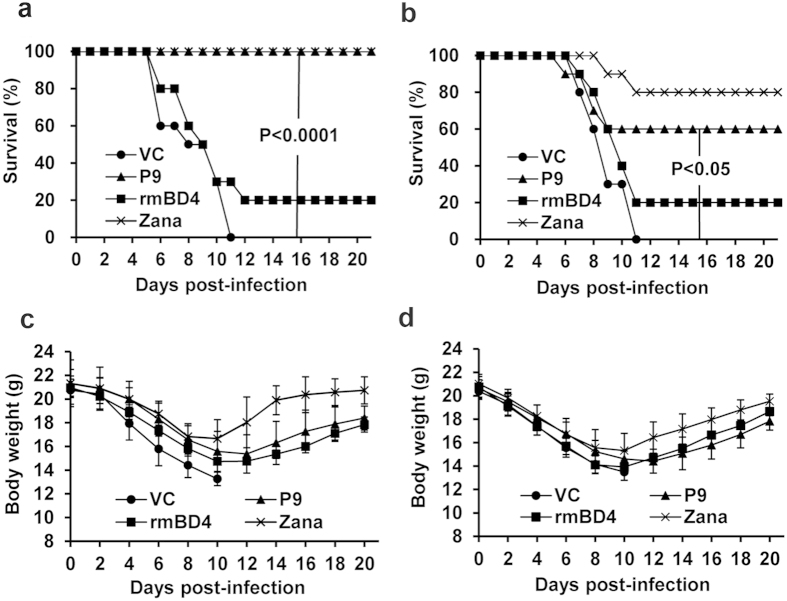
Evaluation of prophylactic and therapeutic effects of P9 in a mouse model. (**a**) Survival rates of mice i.n. inoculated with 25 μl of PB (VC), P9 or rmBD4 (2 mg/ml) before H1N1 virus challenge. (**b**) Survival rates of mice i.n. treated with 25 μl of PB (VC), P9 or rmBD4 (2 mg/ml) after the virus challenge. Zanamivir (Zana, 2 mg/ml) was included as positive control. (**c**,**d**) The body weight of the mice corresponding to (**a**,**b**). Ten animals per group were used for this experiment. *P* values are indicated.

We further evaluated the prophylactic and therapeutic effects of P9 in a lethal mouse model of H1N1 influenza (Fig. 2). The mice were intranasally (i.n.) inoculated with P9 (50 μg/mouse) or rmBD4 and then i.n. challenged with lethal dose of H1N1 virus. The dose of 50 μg/mouse was applied for animal experiments because it was shown to protect all mice from lethal challenge of H1N1 virus. The survival rate of P9-pretreated mice (100%) was significantly higher than those of rmBD4-pretreated mice (20%, *P* < 0.01) and untreated mice (0%, *P* < 0.0001). Notably, pretreatment with P9 and Zanamivir achieved a similar protection effect (Fig. 2a). In parallel, when the mice were i.n. inoculated with 3 doses (50 μg/dose/day) of P9, rmBD4 or Zanamivir at six hours after the lethal challenge with the virus (Fig. 2b), the survival rate in P9-treated mice (60%) were significantly higher than those of untreated control and rmBD4-treated mice (*P* < 0.05). In this setting, treatment of zanamivir yielded a similar level of protection (80%) in comparison to P9 (*P* > 0.05). All infected mice suffered from body weight lost regardless of treatment types. However, the survived mice began to regain body weight at ~10–12 days post-infection (Fig. 2c,d).

**Figure 3:**
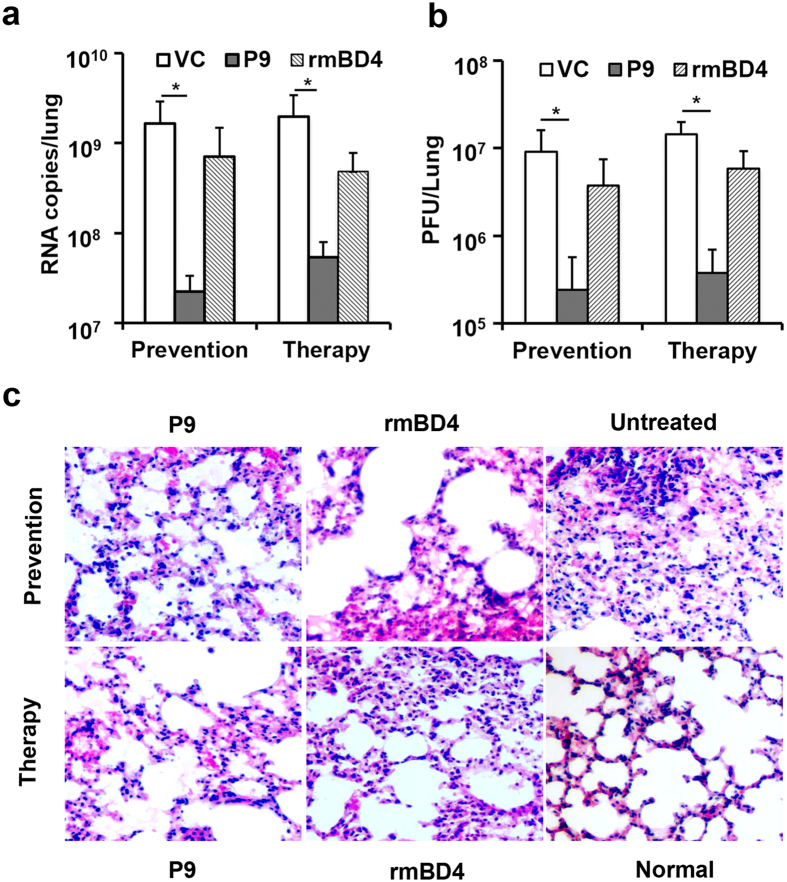
P9 decreased viral loads and pathological changes in lung tissues of infected mice. (**a)** Viral RNA copies in lung tissues of infected mice receiving prophylactic treatment (Prevention) and therapy by i.n. inoculation (Therapy). (**b**) Viral titers in lung tissues of the mice were detected by plaque assay. The results are presented as means + SD of five mice and * indicates *P* < 0.05. (**c**) Histopathological changes in the mouse lung tissues were tested by H&E staining. Representative histological sections of the lung tissues taken from the P9 or rmBD4 treated mice, untreated mice (Untreated) and uninfected mice (Normal) are shown (original magnification 100×).

The viral RNA copies and viral titers in lung tissues of infected mice were detected by real-time RT-PCR (Fig. 3a) and plaque assay (Fig. 3b), respectively. The viral loads in lung tissues of P9-pretreated and P9-treated mice were significantly lower than that of the untreated mice (*P* < 0.05). Histopathological examinations revealed that the alveolar damage and interstitial inflammatory infiltration in untreated mice or mice pretreated or treated with rmBD4 were substantially more severe than those in mice pretreated or treated with P9 (Fig. 3c).

The stability of P9 *in vivo* was evaluated by P9 biodistribution in mouse lungs and its antiviral activity at different time-points after P9 administration. As shown in Supplementary Fig. 2a, P9 could be detected on the surface of mouse bronchial tubs at 10 min, 2 h and 4 h after P9 administration, but the signal decreased to almost undetectable level at 8 h after P9 administration. In the *in vivo* protective experiment, when P9 was administrated to mice at 2 h, 4 h and 8 h before the challenge, it protected 60%, 30% and 20% mice from lethal challenge of H1N1 virus, respectively (Supplementary Fig. 2b). These results indicated that P9 could maintain more than half of its antiviral activity at 2 hours and about 1/5 antiviral activity at 8 hours after administration.

The *in vivo* toxicity of P9 was also assessed in mice. Each mouse was i.n. inoculated with P9 (50 mg/kg) or intra-peritoneally (i.p.) injected with P9 (500 mg/kg) per day for 3 days. As shown in Supplementary Fig. 3, less than 10% of body weight loss was observed in the first 3 days and the body weight began to recover when the treatment was stopped at day 3. There was no obvious reduction of food consumption or sickness during the 10-days observation period. Collectively, our data demonstrated that P9 exhibited prophylactic and therapeutic effects against lethal challenge of H1N1 virus in mice, accompanied with a low toxicity *in vivo*.

### P9 inhibited influenza virus infection through binding to viral surface gylcoproteins

**Figure 4:**
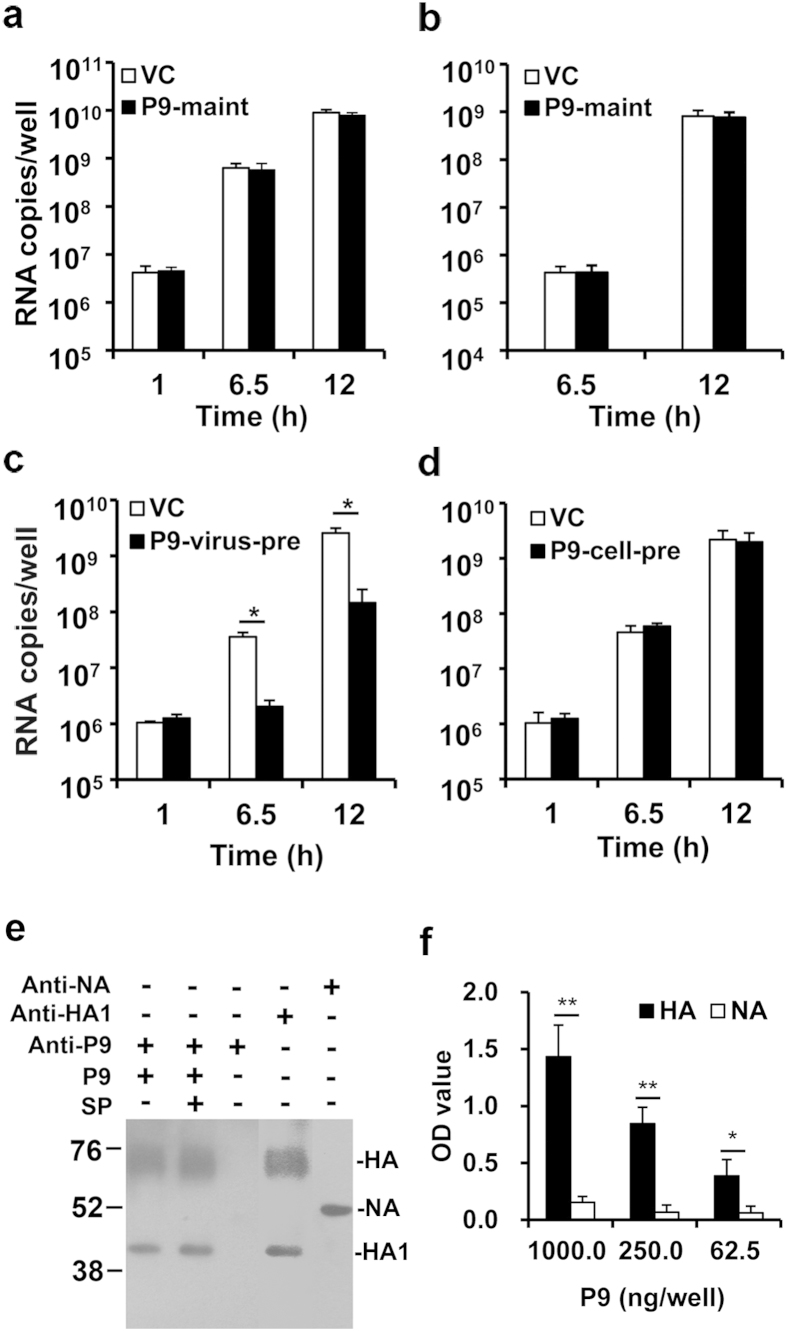
P9 inhibited virus infection by binding to viral glycoprotein. (**a**,**b**) P9 did not inhibit virus replication or release. Viral RNA copies inside cells (**a**) and in supernatants (**b**) of the cultures when P9 was maintained in the culture media after viral infection (P9-maint). (**c**) P9 inhibited the virus infection when the virus was pretreated with P9 (P9-virus-pre). (**d**) P9 did not inhibit viral infection when the cells were pretreated with P9 (P9-cell-pre). Untreated virus controls (VC) were included. All data are presented as means + SD of three independent experiments. * indicates *P* < 0.05. (**e**) P9 bound to viral surface glycoprotein HA of influenza virus. The binding of P9 to H1N1 viral proteins was detected by anti-mBD4 antibody. Scrambled peptide (SP), Anti-HA1 and Anti-NA antibodies were included as controls. (**f**) P9 bound to HA but not NA as determined by ELISA. Data are presented as means + SD of three independent experiments. ** indicates *P* < 0.01.

To investigate the antiviral mechanism of P9, we first seek to determine whether P9 inhibited virus entry, replication, or release. When P9 was supplemented to the culture medium at 1 hour after MDCK cells were infected with H1N1 virus (2 MOI), viral RNA copies inside the cells (Fig. 4a) and in culture supernatants (Fig. 4b) harvested at the indicated hours post-infection were comparable with those in the untreated control (*P* > 0.05). The results suggested that P9 did not inhibit viral replication or release. When the virus was pretreated with P9 and then added to the cells (0.3 MOI), viral loads both inside the cells (Fig. 4c) and in culture supernatants were significantly reduced (Supplementary Fig. 4a,b). In contrast, the viral loads inside the cells (Fig. 4d) and in supernatants (Supplementary Fig. 4c,d) were similar to those of the untreated control when the cells were pretreated with P9 followed by infection. These results indicated that P9 inhibited virus infection by interacting directly with the virus. We further demonstrated that P9 could bind to surface glycoprotein HA of influenza virus (Fig. 4e,f) and S2 of MERS-CoV (Supplementary Fig. 5). Together, our results indicated that P9 inhibited viral infections through binding to viral surface glycoproteins.

### P9 blocked viral RNA release from late endosomes

**Figure 5:**
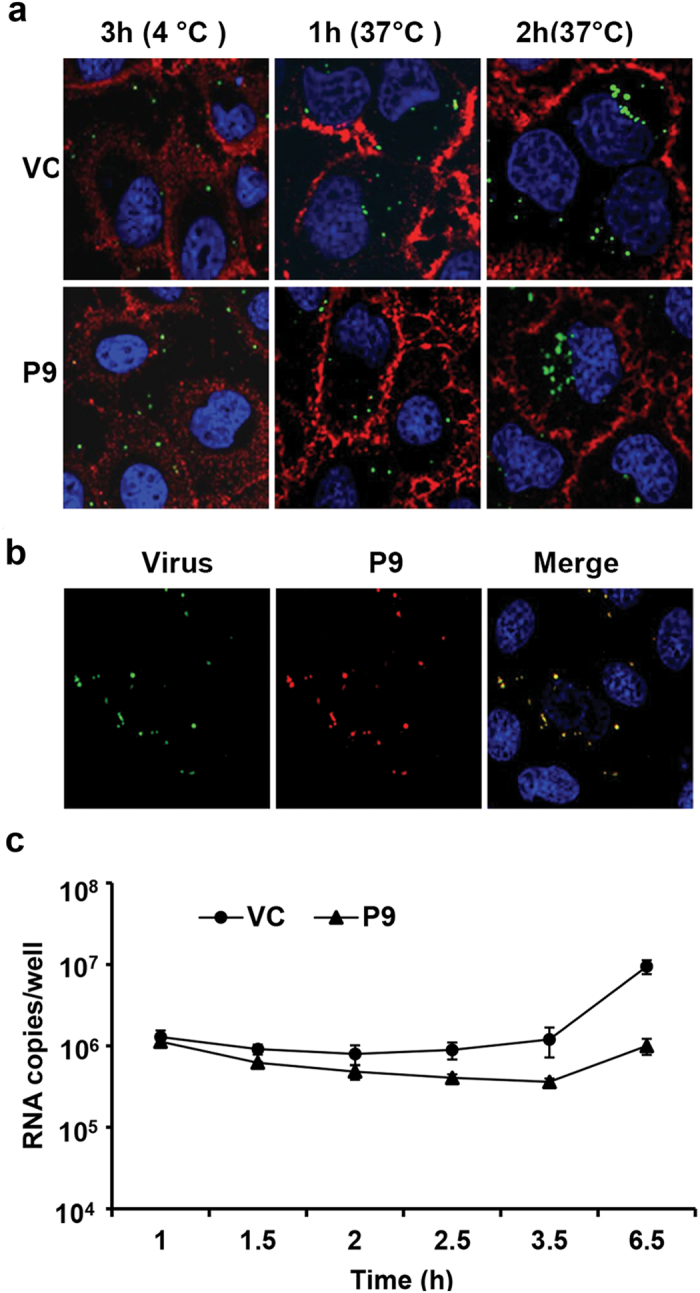
P9 did not inhibit virus-receptor binding or endocytosis but blocked viral RNA release. (**a**) P9 did not inhibit virus-receptor binding or endocytosis. DiO dye labeled virus pretreated with P9 or PB (VC) bound to the cell surface at 4 °C (left side) and entered the cells after incubation at 37 °C for 1 h or 2 h. The cell membrane was stained by Alexa-594 dye and representative images were taken by confocal microscope (original magnification 400×). (**b**) P9 was delivered into the cells by binding to the virus. Colocalization (orange) of virus (green) and P9 (red) was shown after incubation at 37 °C for 2 h. (**c**) P9 blocked the viral RNA replication. Viral RNA copies were detected in the cells infected with the virus pretreated with P9 or PB (VC) at the indicated time points. The results are presented as means ± SD of three independent experiments.

To further dissect how P9 interfered with influenza virus infection, we asked whether virus-receptor binding, endocytosis or viral RNA release was affected by P9. H1N1 virus carrying a green fluorescent label was pretreated with P9 and then incubated with the cells at 4 °C for 3 hours. We found that P9-pretreated virus bound to the cellular membrane in a similar manner compared to the untreated virus (Fig. 5a, left). P9-pretreated virus entered the cells through endocytosis after the virus was incubated at 37 °C for 1 hour (Fig. 5a, middle). Furthermore, the transport of the virus in endosomes from the cell periphery to the proximity of the nucleus was not affected when the virus was pretreated by P9 (Fig. 5a, right). The colocalization of the virus with P9 further confirmed the binding of P9 to the virus, its internalization into cells and its subsequent transport to the perinuclear region together with the virus (Fig. 5b). On the other hand, the levels of viral RNA in infected cells remained essentially unchanged during the first 2 hours post-infection regardless of P9 treatment (Fig. 5c), suggesting that P9 did not inhibit viral binding and entry. The viral RNA levels in cells infected with untreated virus increased at 3.5 hours post-infection, indicative of the initiation of nascent viral RNA replication. In contrast, the viral RNA levels in the cells infected with P9-pretreated virus did not increase until 6.5 hours post-infection and was significantly lower than that of untreated virus control (*P* < 0.05) (Fig. 5c). These results implicated that P9 perturbed viral uncoating and viral RNA release from late endosomes for subsequent replication.

### P9 prevented endosomal acidification

**Figure 6:**
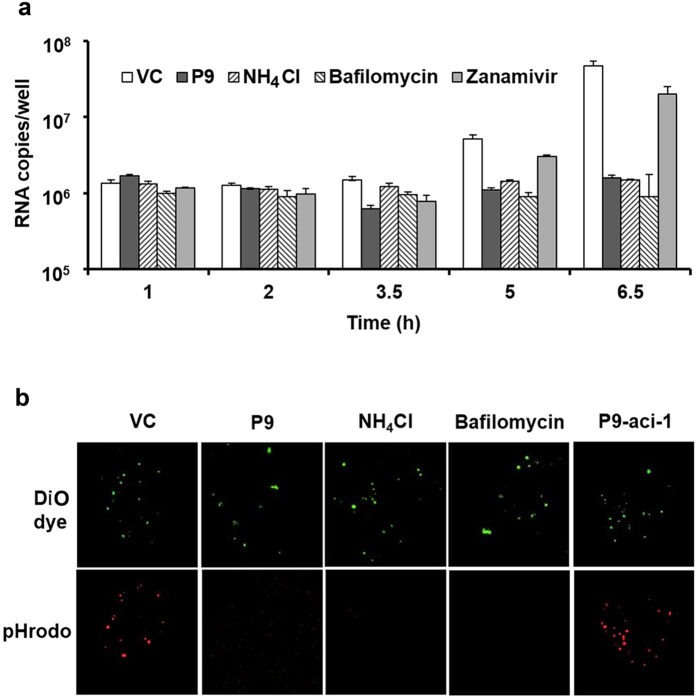
P9 suppressed pH lowering in endosomes. (**a**) The inhibitory effect of P9 was similar to those of inhibitors of endosomal acidification. Viral RNA copies in the cells infected with the virus pretreated with PB (VC), P9, NH_4_Cl or Bafilomycin were measured at the indicated time-points post-infection. NH_4_Cl and Bafilomycin were maintained in the culture medium after viral infection. The results are presented as means + SD of three independent experiments. (**b**) P9 inhibited pH decrease in endosomes. Cells were infected with DiO dye labeled virus (green) pretreated with PB (VC), P9, NH_4_Cl, Bafilomycin or P9-aci-1. The low pH in endosomes of infected cells was stained by pH-sensitive dye (pHrodo, red). Representative images were taken by confocal microscope (original magnification 400×).

Endosomal acidification is a prerequisite for influenza virus uncoating and the subsequent genome release. Bearing in mind the basic amino acid-rich composition of P9, we examined whether P9 could block viral RNA release by counteracting endosomal acidification. As shown in Fig. 6a, P9 showed similar antiviral effects in comparison to ammonium chloride (NH_4_Cl) and Bafilomycin A1, which are well-defined inhibitors of endosomal acidification^21^,^22^,^23^. In contrast, Zanamivir, which inhibits viral release, did not show a similar antiviral kinetics in comparison to the endosomal acidification inhibitors. Therefore, P9, NH_4_Cl and Bafilomycin A1 might share a similar antiviral mechanism of inhibiting pH decrease in endosomes. To confirm this hypothesis, we detected the pH change in endosomes using a pH-sensitive dye, the intensity of which mirrored the acidity of the cellular milieu. Red signals in the cells infected with untreated virus (VC) indicated the acidification process in endosomes (Fig. 6b). In contrast to the control, when the cells were infected with virus pretreated with P9, NH_4_Cl or Bafilomycin A1, little to no any red signals were observed, indicating that acidification was abolished in the endosomes. To verify this, we designed a P9 analog named P9-aci-1 containing three additional acidic amino acids at the C-terminus (Table 1). If P9 acts through perturbation of pH lowering, the addition of acidic residues is expected to relieve the perturbation and abrogate the consequent antiviral effect. Indeed, confocal microscopic images indicated that pH lowering in endosomes was unaffected by P9-aci-1 (Fig. 6b). In line with the imaging result, P9-aci-1 lost its antiviral activity (IC_50_ > 100 μg/ml). In addition, the polykaryon assay further showed that P9 did not directly inhibit HA-cell membrane fusion (Supplementary Fig. 6). Hence, when delivered into the endosomes, virus-bound P9 prevented pH decrease, resulting in the inhibition of viral RNA release and subsequent replication.

### P9 showed broad-spectrum antiviral effects against respiratory viruses *in vitro* and *in vivo*

**Figure 7:**
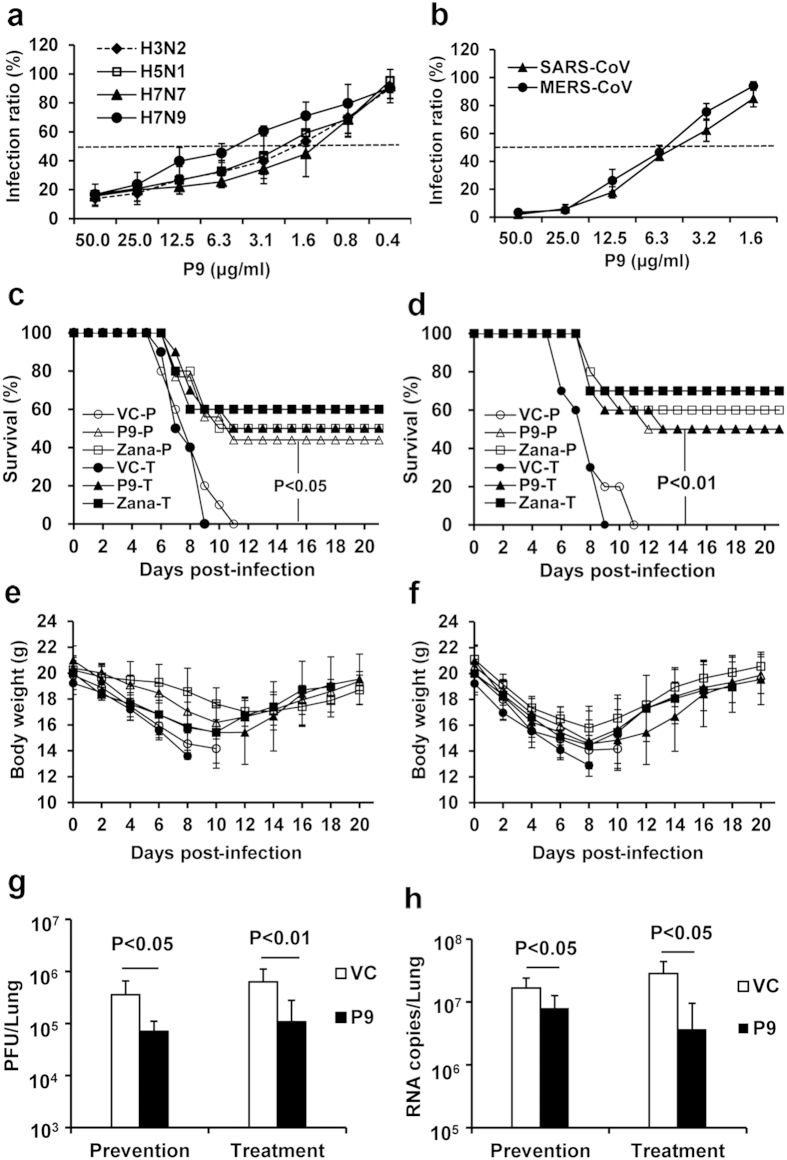
Detection of antiviral effects of P9 against infections of multiple respiratory viruses. (**a**) P9 inhibited infections of influenza virus subtypes H3N2, H5N1, H7N7 and H7N9 in cells. (**b**) P9 inhibited infections of SARS-CoV and MERS-CoV in cells. IC_50_s are indicated by dotted lines. The results are presented as means ± SD of three independent experiments. (**c**) P9 protected mice from lethal challenge of H5N1 virus. (**d**) P9 protected mice from lethal challenge of H7N9 virus. (**e** and **f**) Body weight of the mice corresponding to (**c**,**d**). (**g**,**h**) P9 inhibited the infection of SARS-CoV in mice. Lung tissues of infected mice were collected at day 3 post-infection. Viral titers in lung tissues were detected by plaque assay (**g**) and real-time RT-PCR (**h**). To evaluate prophylactic effect of P9, mice were intratracheally (i.t.) inoculated with 50 μl of PB (VC-P), Zanamivir (Zana-P) or P9 (P9-P). To evaluate therapeutic effect of P9, mice were i.n. treated by PB (VC-T), Zanamivir (Zana-T) or P9 (P9-T) after viral challenge. *P* values are indicated.

We next evaluated whether P9 could inhibit other subtypes of influenza virus and other respiratory viruses such as SARS-CoV and MERS-CoV, because they all depend on endosomal acidification for viral infection. Our data demonstrated that P9 exhibited strong antiviral effects against other subtypes of influenza A virus including H3N2, H5N1, H7N7 and H7N9 with IC_50_s ranging from 1.5 to 4.8 μg/ml (Fig. 7a). The IC_50_s of P9 against SARS-CoV and MERS-CoV were about 5 μg/ml. Notably, at concentrations higher than 25 μg/ml, P9 could inhibit SARS-CoV and MERS-CoV infections to more than 95% (Fig. 7b). These results indicated that P9 has broad-spectrum antiviral activities *in vitro* against multiple respiratory viruses.

We further evaluated the protective effect of P9 against infections of H5N1, H7N9 and SARS viruses in mice. As shown in Fig. 7c, one dose (100 μg/mouse) of P9 for prophylaxis (P9-P) and 5 doses (100 μg/mouse) of P9 for therapy (P9-T) could protect 44% and 50% of mice from lethal challenge of H5N1 virus, respectively. These protection rates were significantly higher than that of untreated mice (*P* < 0.05) and comparable with the mice treated with prophylactic and therapeutic Zanamivir (Zana; 50% and 60%, *P* > 0.05). One dose of P9 for prophylaxis and 5 doses of P9 for therapy protected 50% and 50% of mice from lethal challenge of H7N9 virus, respectively. The treatment groups provided significantly more protection than the control group (*P* < 0.01). The survival rates of mice treated with prophylactic and therapeutic Zanamivir were 60% and 70%, respectively, which did not show significant difference compared to those of P9-treated mice (*P* > 0.05) (Fig. 7d). The body weight of all treated and untreated mice began to decrease at day 2 post-challenge and then gradually recovered from day 12 post-infection in the survival mice (Fig. 7e,f). Since we have not established animal models for lethal infection of MERS-CoV and SARS-CoV, we compared the viral load in mouse lungs at day 3 post-infection in a mouse model of non-lethal SARS-CoV infection. As shown in Fig. 7g,h, one dose of P9 for prophylaxis and 5 doses of P9 for therapy significantly inhibited virus infection in mouse lungs compared to that in the untreated mouse lungs (*P* < 0.05). These results demonstrated that P9 could provide broad-spectrum protection *in vivo* against infections of multiple respiratory viruses.

## Discussion

In this study, we found that a short peptide, P9, exhibited the highest antiviral activity against influenza A virus H1N1 among a panel of mBD4-derived peptides, smBD4, and rmBD4 (Fig. 1). At the same time, P9 showed the lowest cytotoxicity *in vitro*, resulting in an improved selectivity index over the parental rmBD4 and smBD4. It was particularly noteworthy that P9 displayed potent prophylactic and therapeutic effects in mice (Fig. 2). The production of recombinant BDs is hampered by high cost and low recovery rate (Supplementary Fig. 7). In contrast, P9 can be chemically synthesized in high yield and with low cost. Synthetic P9 is highly soluble and stable in water, as well as reasonably stable *in vivo*, making P9 an ideal lead compound for further development as a novel antiviral drug.

It has been reported that the antiviral activity of BDs might be mediated through either indirect interaction or direct interaction with viral glycoproteins and/or envelopes^14^,^15^. We here demonstrated the binding of P9 with viral glycoproteins (Fig. 4e,f and Supplementary Fig. 5), but not the target cells (Fig. 4d). The infection of influenza virus involves multiple steps including virus-receptor binding, endocytosis, transport from early endosomes to late endosomes where acidification results in virus-endosome membrane fusion, subsequent viral disassembly (uncoating), and viral RNA release, which triggers viral RNA replication^12^,^22^. We demonstrated that P9 might mitigate viral RNA release from late endosomes to inhibit viral RNA replication (Fig. 5). Since pH lowering to 5.0 in late endosomes is critical for virus-endosome membrane fusion^22^,^24^, inhibitors of endosomal acidification can efficiently inhibit membrane fusion by suppressing the pH decrease in endosomes^21^,^22^,^25^. P9 was indeed capable of inhibiting the pH decrease in endosomes in a similar fashion comparing to two well-characterized inhibitors of endosomal acidification, NH_4_Cl and Bafilomycin A1 (Fig. 6). Thus, our data suggested that P9 inhibited viral RNA release by suppressing pH decrease in endosomes. The antiviral function of P9 mediated through inhibition of endosomal acidification might be independent of the blocking of M2 ion channel, since P9 could efficiently inhibit the infection of three amantadine-resistance strains of influenza virus, A/Hong Kong/415742/2009 (H1N1), A/Vietnam/1194/2004 (H5N1) and A/Anhui/1/2013 (H7N9) (Fig. 7)^26^,^27^,^28^.

Importantly, for a substantial number of pathogenic viruses, including influenza virus and coronavirus, virus-endosome membrane fusion is a prerequisite for viral disassembly and viral genome release into host cells to initiate productive viral replication^4^,^29^. Membrane fusion is mediated by viral envelope glycoproteins and requires an acidic environment in endosomes^4^,^30^. In line with the antiviral mechanism of P9 illustrated in this study, P9 could inhibit infections of other subtypes of influenza A virus including H3N2, H5N1, H7N7 and H7N9, as well as two coronaviruses, SARS-CoV and MERS-CoV (Fig. 7). Our results also demonstrated that P9 could bind to S2 of MERS-CoV (Supplementary Fig. 5), suggesting that the antiviral mechanism of P9 against SARS-CoV and MERS-CoV was potentially similar to that against influenza virus. We reasoned that P9 might be capable of inhibiting the infection of additional pathogenic viruses, which enter target cells via endocytosis, as long as P9 could efficiently bind to the viral particles.

In summary, we have demonstrated that a short peptide derived from mBD4 has broad-spectrum antiviral activity against different subtypes of influenza A virus including H1N1, H3N2, H5N1, H7N7 and H7N9, as well as two coronaviruses, SARS-CoV and MERS-CoV. The antiviral effect of P9 is mediated through the following mechanism: (1) P9 efficiently binds to viral particles via viral glycoproteins and (2) P9 is rich in basic amino acids, which prevent acidification in endosomes and inhibit viral RNA release. Influenza A virus and coronavirus have caused several fatal pandemics and outbreaks in the past century. Although a number of anti-influenza drugs have been developed, drug-resistant virus strains emerged quickly after these antiviral drugs were put in clinical use. In addition, thus far, no effective antiviral drugs are available for patients infected with SARS-CoV or MERS-CoV^5^. In this regard, antiviral peptides, such as P9, represent new promising prophylactic and therapeutic agents with broad-spectrum antiviral activities. Overall, based on the mechanism illustrated in this study, new antiviral peptides with broad-spectrum antiviral activities may be developed to provide new drugs for prophylaxis and therapy of viral infections.

## Methods

### Viruses and cell cultures

A highly virulent mouse adapted influenza A virus mutant strain A/Hong Kong/415742Md/2009 (H1N1)^31^ as well as other influenza A virus strains A/Hong Kong/415742/2009 (H1N1), A/Hong Kong/8/68 (H3N2), A/Vietnam/1194/2004 (H5N1), A/Netherlands/219/2003 (H7N7) and A/Anhui/1/2013 (H7N9) were cultured in Madin-Darby canine kidney (MDCK) cells and the titers were determined by plaque and TCID_50_ assay as described previously^32^. SARS-CoV strain HKU39849 and MERS-CoV strain hCoV-EMC/2012 were cultured in fetal rhesus monkey kidney (FRhK-4) and African green monkey kidney E6 (Vero-E6) cells and their titers were determined by plaque and TCID_50_ assays as described previously^33^.

### Cloning, expression and purification of rmBD4

The codons of mBD4 were optimized to *E. coli*-preferred codons based on OPTIMIZER^34^ (Supplementary Fig. 7a). The fragment of mBD4 was generated by PCR-based gene synthesis using 6 oligos (Supplementary Fig. 7b). The codon-optimized gene was cloned into PCR2.1 vector and then sub-cloned into PET32a(+) at KpnI and XhoI sites. The resulting plasmid was transformed into BL21 (DE3) to express thioredoxin-mBD4 fusion protein. Thioredoxin-mBD4 (Trx-mBD4) was released from *E. coli* by a simple osmotic shock procedure^35^ and further purified by AKTA-FPLC (GE Healthcare, Little Chalfont, Buckinghamshire, United Kingdom) using His Trap FF column (GE Healthcare) (Supplementary Fig. 7c). Trx-mBD4 was digested with enterokinase to release recombinant mBD4 (rmBD4). rmBD4 was recovered by cation-exchange chromatography using Sp sepharose FF (GE Healthcare). Purified rmBD4 was desalted using PD-10 column (GE Healthcare) into 25 mM HEPES buffer (pH 7.4) (Supplementary Fig. 7d).

### Peptide design and evaluation of antiviral effects

Full-length mBD4 and short peptides derived from mBD4 were designed as shown in Table 1 and synthesized by ChinaPeptide (Shanghai). The purity of all peptides was >99%. The purity and mass of each peptide were verified by HPLC and mass spectrometry. Antiviral effects of the short peptides, smBD4 and rmBD4, were initially evaluated in a low-salt medium, i.e. 30 mM phosphate buffer (PB) containing 24.6 mM Na_2_HPO_4_ and 5.6 mM KH_2_PO_4_ pH 7.4^36^ and in a high-salt minimum essential medium (MEM). Peptides (0.4–100.0 μg/ml) were premixed with H1N1 virus in PB or MEM and incubated at room temperature for 1 hour. MDCK cells were infected by peptide-pretreated or untreated virus, while Zanamivir (0.2–10.0 μg/ml) was maintained in culture medium as positive control. Antiviral activity of the peptides was measured using a plaque reduction assay as described previously^37^. The infection ratio was calculated as the plaque number of virus pretreated with peptides being divided by the plaque number of virus pretreated with PB or MEM.

### Cytotoxicity and IC_50_ assays

Cytotoxicity of peptides was determined by the detection of 50% toxic concentration (TC_50_) using a tetrazolium-based colorimetric (MTT) assay as described previously^38^. Briefly, we exchanged the solution buffer of all peptides with distilled water and concentrated them by vacuum dryer at −40 °C for toxicity assay. MDCK cells were seeded in 96-well cell culture plate at an initial density of 1 × 10^4^ cells/well in 100 μl of MEM supplemented with 10% FBS and incubated for overnight. The cells were washed twice with PBS then added with 100 μl/well of MEM with various concentrations of peptides. Experiments were done in triplicates. After incubation at 37 °C for 24 hour, MTT solution (5 mg/ml, 10 μl/well) was added to the plate. After the plate was incubated at 37 °C for 4 hours, 100 μl of 10% SDS in 0.01 M HCl was added to each well. After further incubation at 37 °C overnight, the plate was read at OD_570_ and OD_640_ using Victor^TM^ X3 Multilabel Reader (PerkinElmer). Cell culture wells without peptides were used as the experiment control and medium only served as a blank control. IC_50_s of peptides against different subtypes of influenza A virus, i.e. H1N1, H3N2, H5N1, H7N7 and H7N9, and coronaviruses SARS-CoV and MERS-CoV were calculated from the infection ratio of virus treated by various concentrations of peptides^32^.

### Evaluation of antiviral effects *in vivo*

BALB/c female mice, 6–8 weeks old, were kept in biosafety level 3 laboratory and given access to standard pellet feed and water *ad libitum*. All experiments were carried out in accordance with the approved guidelines of the biosafety level 3 animal facilities and were approved by the Committee on the Use of Live Animals in Teaching and Research of the University of Hong Kong^32^. The mouse adapted H1N1, H5N1, and H7N9 viruses were used for lethal challenge of mice. To evaluate the prophylactic effects, mice were i.n. or i.t. inoculated with PB, P9, rmBD4, or Zanamivir, and then challenged with 3 LD_50_ of the virus at 10 min later. For evaluation of the therapeutic effect, mice were challenged with 3 LD_50_ of the virus and then i.n. inoculated with P9, rmBD4, or Zanamivir at six hours after the viral inoculation. Two more doses were given to H1N1-challenged mice and four more doses were given to H5N1- and H7N9-challenged mice at 1-day interval. Survival and general conditions were monitored for 21 days or until death. For virological and pathological tests, 3 mice in each group were sacrificed at day 5 after challenge. To evaluate *in vivo* prophylactic effect of P9 against SARS-CoV infection, nine-month old mice were i.t. inoculated with P9 (100 μg/mouse) and then challenged with 2 × 10^4^ TCID_50_ of SARS-CoV. For evaluation of therapeutic effect, mice were challenged with 2 × 10^4^ TCID_50_ of SARS-CoV and then i.n. inoculated with P9 at 6 hours post-infection. Four more doses were given to the mice in the following two days. Lung tissues were collected at day 3 post-infection for virological assay. All i.t. inoculations were performed using a MicroSprayer Aerosolizer (Penn-Century, USA). At least nine mice in each group were analyzed in all animal experiments.

### Histopathological staining

Lung tissues collected from the challenged mice were immediately fixed in 10% buffered formalin, applied to dehydration and embedded in paraffin wax. Sections of 5 μm thickness were mounted on slides. Histopathological changes were examined by hematoxylin and eosin (H&E) staining under a light microscope as described previously^32^.

### Viral RNA extraction and real-time RT-PCR

Viral loads were detected by real-time RT-PCR as described previously^31^. Viral RNA was extracted from culture supernatants using RNeasy Mini Kit (Qiagen), while viral RNA in cell lysis and mouse lung tissues was extracted using QIAamp Viral RNA Mini Kit (Qiagen), according to the manufacturer’s protocols. Reverse transcription was performed using RSII kit (Invitrogen) according to the manufacture’s instruction. Real-time PCR was performed using ABI SYBR Green Mastermix and the 7500 system. The clone of HA gene of H1N1 virus served as the positive control and standard. Real-time PCR experiments were performed in triplicate.

### Fluorescence imaging

The H1N1 virus was labeled with green fluorescent lipophilic dye DiO (Invitrogen, 3898), while P9 was detected by 1:5000 diluted primary antibody rabbit-anti-smBD4 (Max Biotechnology Co. Ltd., China) and 1:400 diluted secondary antibody goat anti-rabbit Alexa 594 (Invitrogen, A11012). The cell membrane was stained with dye Alexa 594 (Invitrogen, W11262). The labeled virus and P9 were premixed for 1 hour and then incubated with MDCK cells at 4 °C or 37 °C. The infected cells were fixed by 4% formalin at different time points after infection. After cells were permeablized by 0.1% Triton X-100 and reacted with corresponding antibodies, images were taken under confocal microscope (Carl Zeiss LSM 780).

### Polykaryon assay

293FT cells were seeded into 12-well plates at 4 × 10^5^ cells per well. After 16 hours of culture, the cells were transfected with phw2000-HA plasmid (0.8 μg/well) using Lipofectamine 3000 (Life Technologies) following the manufacture’s instruction. At 24 hours after the transfection, the transfection medium was replaced by plain DMEM and cells were treated by trypsin (1 μg/ml) for 10 min at 37 °C. After rinsing the cells with plain DMEM, the cells were pre-incubated with P9 or 0.3% DMSO in DMEM for 20 min at 37 °C. Polykaryon formation was induced by exposing the cells to low pH DMEM (pH 5.0) containing the corresponding concentration of P9 or DMSO for 10 min at 37 °C. The low pH medium was replaced with DMEM containing 10% FBS and the cells were incubated for 3 h at 37 °C. Finally, the cells were fixed with 10% buffered formalin, stained with Giemsa (Sigma). The images were taken by microscope at 200 × magnification.

### Detection of the endosomal acidification

Endosomal acidification after viral infection was detected according to the instructions provided by the manufacturer of pH-sensitive dye (pHrodo Red dextran, Invitrogen, P10361). H1N1 virus was pre-labeled with DiO and then incubated with peptides, NH_4_Cl, Bafilomycin A1, or PB (untreated) at room temperature for 45 min, followed by incubation at 4 °C for 15 min. MDCK cells were inoculated with 10 MOI of P9-treated or untreated virus and incubated at 4 °C for 1 hour, then 100 μg/ml of pH-sensitive dye was added to the cells and the incubation was continued at 4 °C for 10 min. Before taking image, cells were further cultured at 37 °C for 10 min and then the cells were washed two times with PBS. Finally, fresh media were added to cells and images were taken immediately under confocal microscope (Perkin Elmer Spinning Confocal Microscope).

### Western blot assay

Viral protein bound with P9 was identified by Western blot assay as described previously^39^. Briefly, viral protein samples were separated by SDS-PAGE and transferred to the polyvinylidene difluorid (PVDF) membrane. The transferred membrane was incubated with P9 (2 μg/ml) at room temperature for 1 h, followed by incubation with rabbit anti-smBD4 (1:5000) for 1 h to detect P9 binding. For detection of HA, NA, S1, and S2, the membrane was incubated with rabbit-anti-HA1 (1:4,000, Immune Technology Crop., IT-003-001), or rabbit-anti-NA (1:4,000, Immune Technology Crop., IT-003-038), rabbit-anti-S1, (1:4000, Sino Biological Inc. 100209-RP02) or rabbit-anti-S2 (1:4000, Sino Biological Inc. 100210-RP02) at room temperature for 1 h. Goat anti-rabbit IgG-HRP (1:10,000, Invitrogen, 656120) was used as the secondary antibody to detect the binding at room temperature for 1 h. After the membrane was probed with the indicated antibodies or the peptide, immunoreactive bands were visualized by enhanced chemiluminescence.

### ELISA

Binding affinities of the peptides to viral protein were detected by ELISA as described previously^40^ with some modification. Briefly, various concentrations of peptides were coated to ELISA plates and incubated with blocking buffer at 4 °C overnight. After addition of H1N1 viral HA, NA, MERS-CoV S1, or S2 (Sino Biological Inc.) and incubation at 37 °C for 1 hour, the binding affinities of the peptides to viral protein were determined by rabbit anti-His (1:2,000, Santa Cruz Biotechnology Inc, SC-8036). Goat anti-rabbit IgG-HRP was used as the secondary antibody (1:4,000, Invitrogen, 656120). Readings were obtained in an ELISA reader (Victor 1420 Multilabel Counter; PerkinElmer).

### Statistical Analysis

Survival of mice and the statistical significance were analyzed by GraphPad Prism 5. The statistical significance of the other results was calculated by the two-tailed Student *t* test using Stata statistical software. Results were considered significant at *P* < 0.05.

## Additional Information

**How to cite this article**: Zhao, H. *et al.* A novel peptide with potent and broad-spectrum antiviral activities against multiple respiratory viruses. *Sci. Rep.* **6**, 22008; doi: 10.1038/srep22008 (2016).

## Supplementary Material

Supplementary Information

## Figures and Tables

**Table 1:**
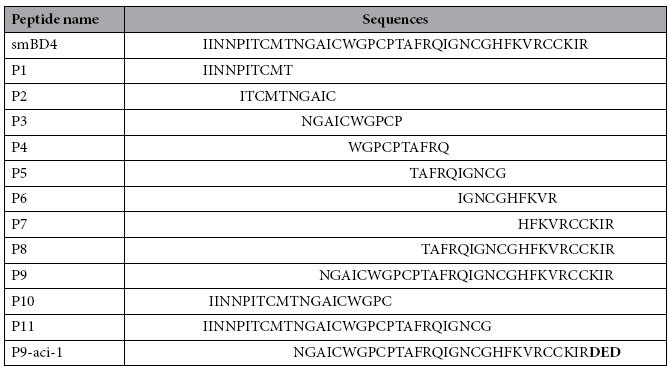
Sequences of peptides derived from mBD4.

Note: P9-aci-1: three acidic amino acids D, E and D were added to the C-terminus of P9.
