# Supplementary material for: A novel peptide with potent and broad-spectrum antiviral activities against multiple respiratory viruses
Source: Sci Rep. 2016 Feb 25;6:22008. doi: 10.1038/srep22008 (PMC4766503; doi:10.1038/srep22008)
Supplement: Supplementary Information [file srep22008-s1.doc]

**Supplementary information:**

**A novel peptide with potent and broad-spectrum antiviral activities against multiple respiratory viruses**

Hanjun Zhao^1^, Jie Zhou^1^, Ke Zhang^1^, Hin Chu^1^, Dabin Liu^1^, Vincent Kwok-Man Poon^1^, Chris Chung-Sing Chan^1^, Ho-Chuen Leung ^1^, Ng Fai^1^, Yong-Ping Lin^1^, Anna Jin-Xia Zhang^1^, Dong-Yan Jin^2^, Kwok-Yung Yuen^1^, Bo-Jian Zheng^1*^

**Supplementary Figures**


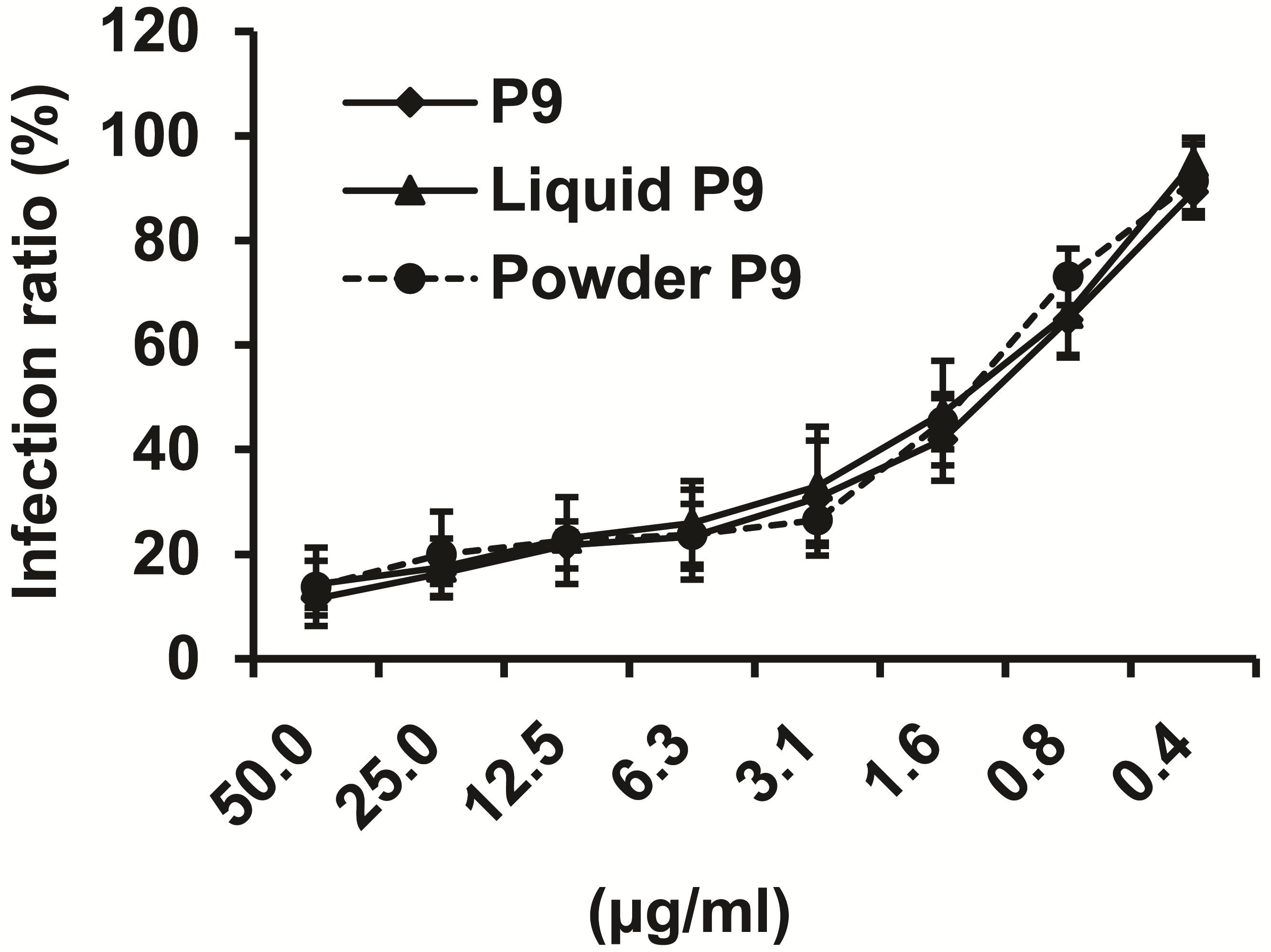


**Figure 1. The stability of P9 stored at -20°C for one year in liquid and powder form.** The antiviral activity of fresh P9, stored liquid P9 and powder P9 was detected by plaque reduction assay.


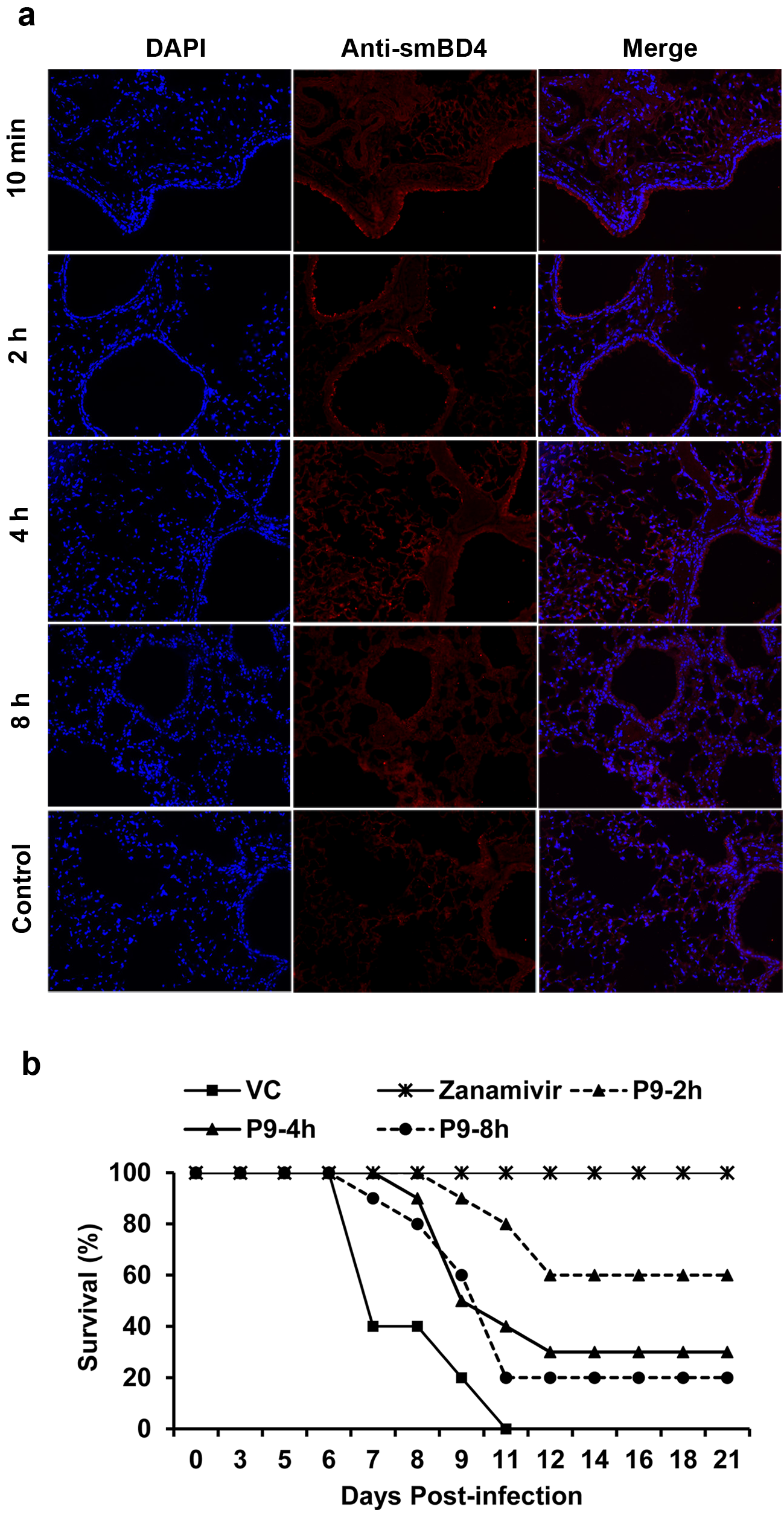


**Figure 2. The stability of P9 *in vivo*. (a)** The biodistribution of P9 in mouse lung tissues was detected at the indicated time-points after P9 (50μg/mouse) was i.t. inoculated to mouse lungs. Lung tissues from normal mice without P9 inoculation were included as negative control (Control). P9 was recognized by rabbit anti-smBD4. The signals were expressed by goat anti-rabbit Alexa 594. Representative images were taken by microscope (Original magnification 200×). (**b**) The prophylactic efficiency of P9 at different time points before H1N1 virus challenge. Mice (10/group) were challenged with lethal dose of H1N1 virus at 2h, 4h and 8h after they were i.t. inoculated with P9. Two groups of mice were i.t. inoculated with PB buffer (VC) and Zanamivir at 2h before the virus challenge as negative and positive controls, respectively. Survival of mice was monitored for 21 days or till the mouse death.


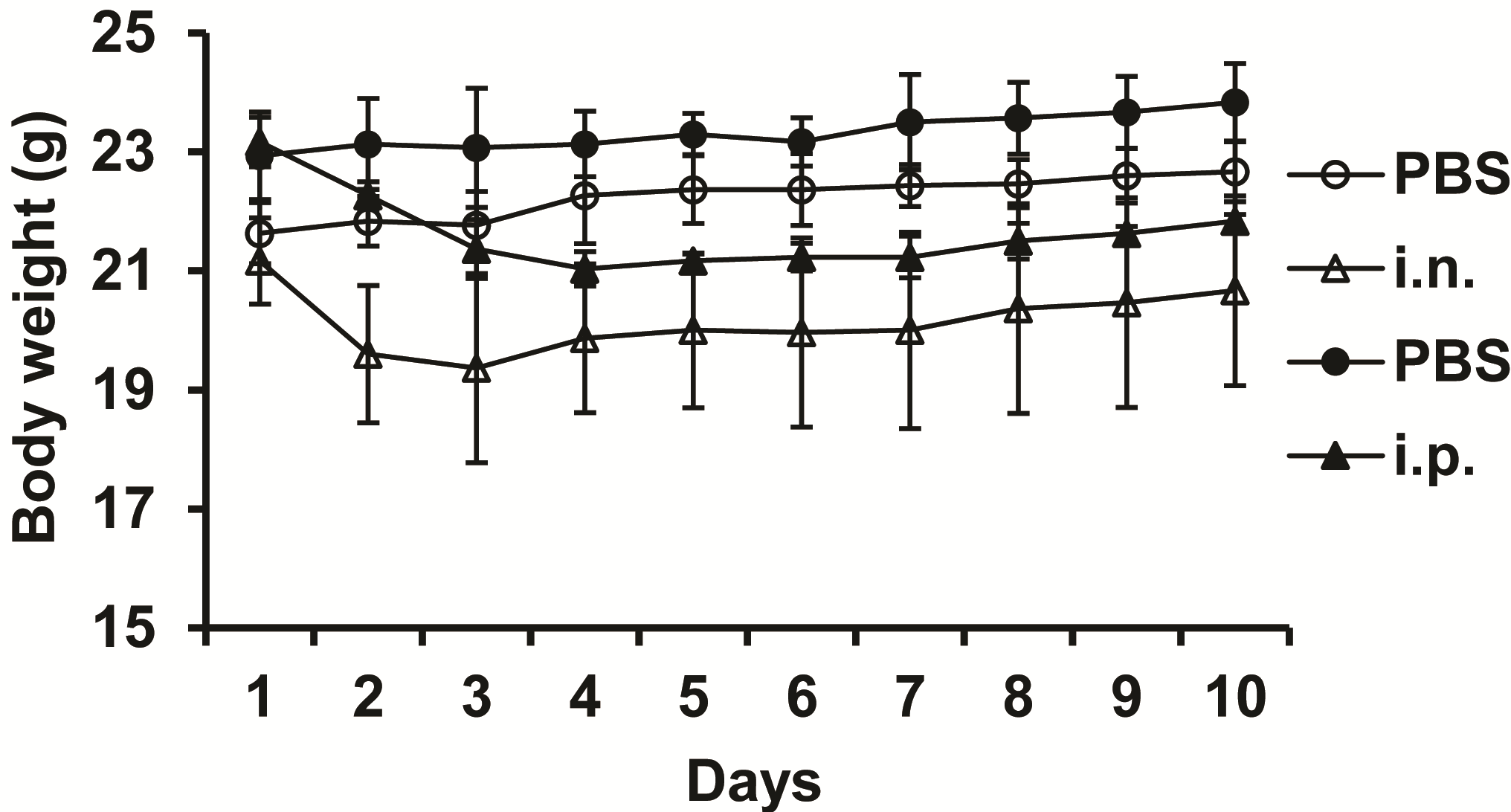


**Figure 3. The *in vivo* toxicity of P9.** P9 was intranasally (i.n.) inoculated to mouse lungs or intra-peritoneally (i.p.) injected to the mice. PBS was i.n. or i.p. inoculated to the mice as the negative control. Body weights of the mice were measured daily for 10 days. The results are presented as means ± SD of five mice.


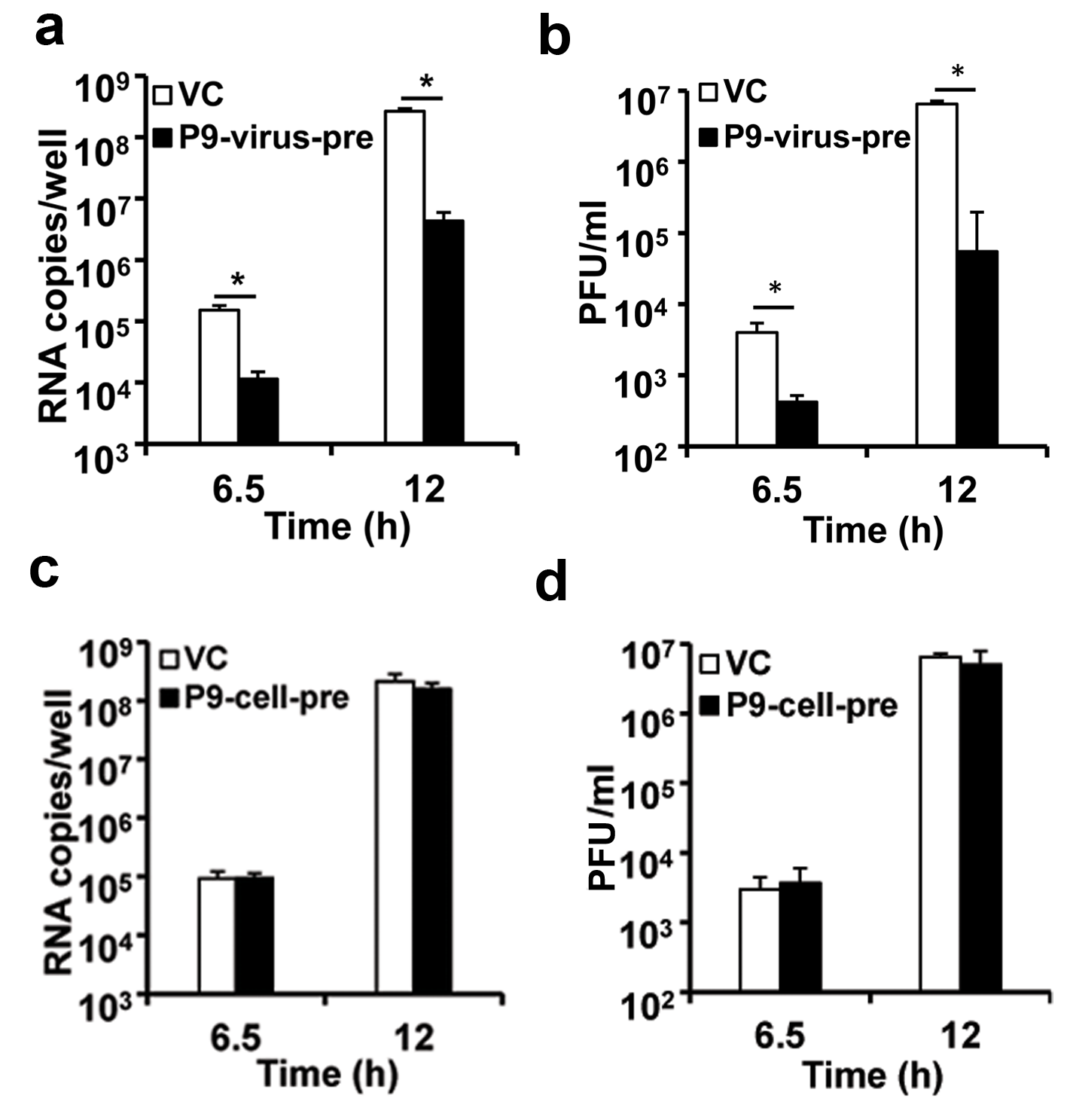


**Figure 4. P9 inhibited the viral infection in MDCK cells via interaction with the virus.** (**a** and **b**) P9 inhibited the viral infection via interaction with the virus. The virus was pretreated with P9 (P9-virus-pre) or PB (VC) and then infected the cells. Viral RNA copies (**a**) and virus titers (**b**) in culture supernatants were detected by real-time RT-PCR and plaque assay, respectively. (**c** and **d**) P9 did not inhibit the viral infection via interaction with target cells. MDCK cells were pretreated with P9 (P9-cell-pre) or PB (VC) and then infected with H1N1 virus. Viral RNA copies (**c**) and virus titers (**d**) in culture supernatants were detected by real-time RT-PCR and plaque assay, respectively. Data are presented as means + SD of three independent experiments and * indicates *P* < 0.05.


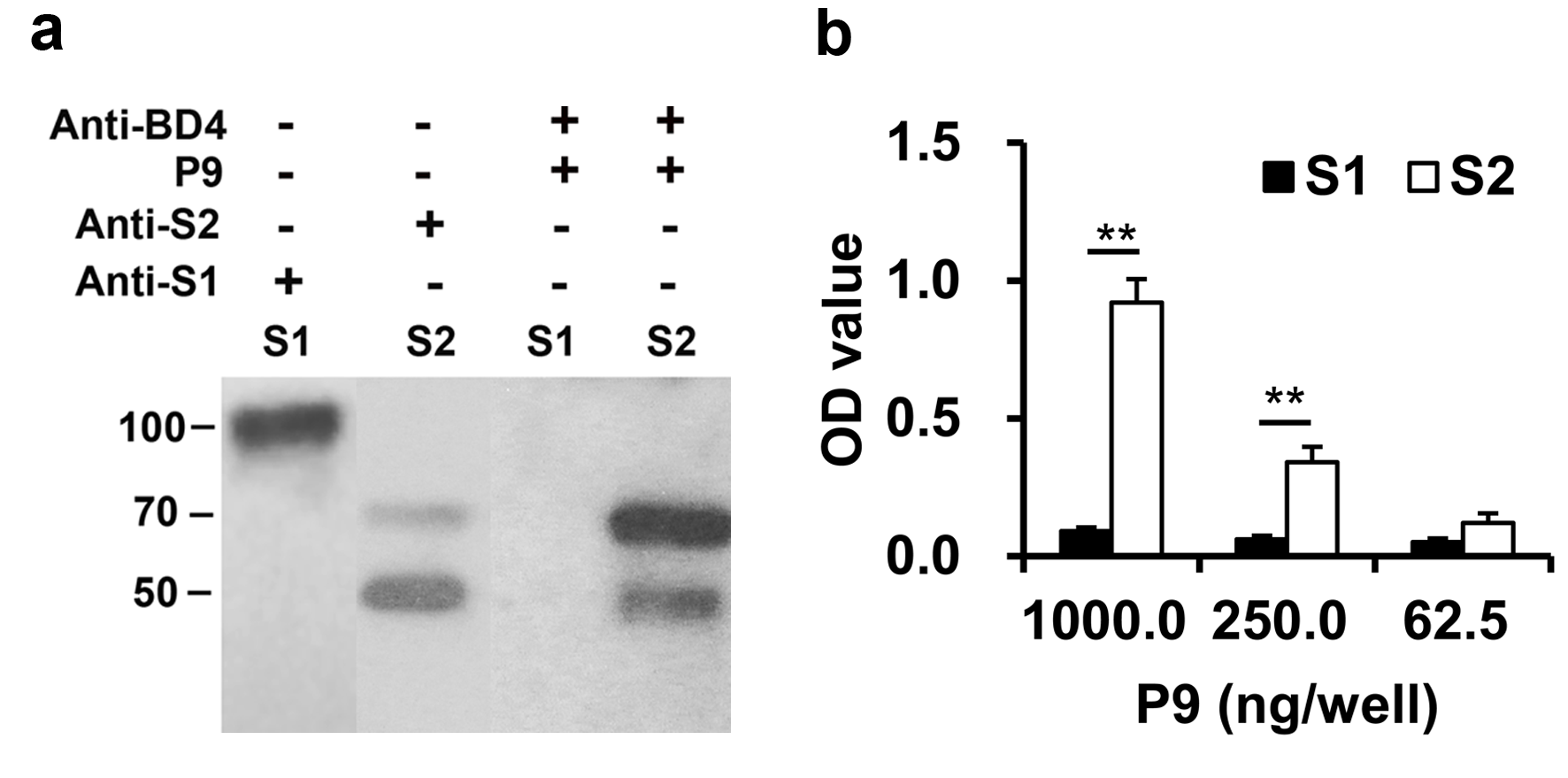


**Figure 5. P9 bound to spike protein S2 of MERS-CoV.** (**a**) P9 bound to viral glycoprotein S2 as determined by Western blot assay. The specific binding of P9 to spike protein S1 and S2 of MERS-CoV was detected by anti-mBD4 antibody. Anti-S1 and Anti-S2 antibody were included as controls. (**b**) P9 bound to S2 but not S1 as determined by ELISA. Data are presented as mean+SD of three independent experiments. ** indicates *P*<0.01.


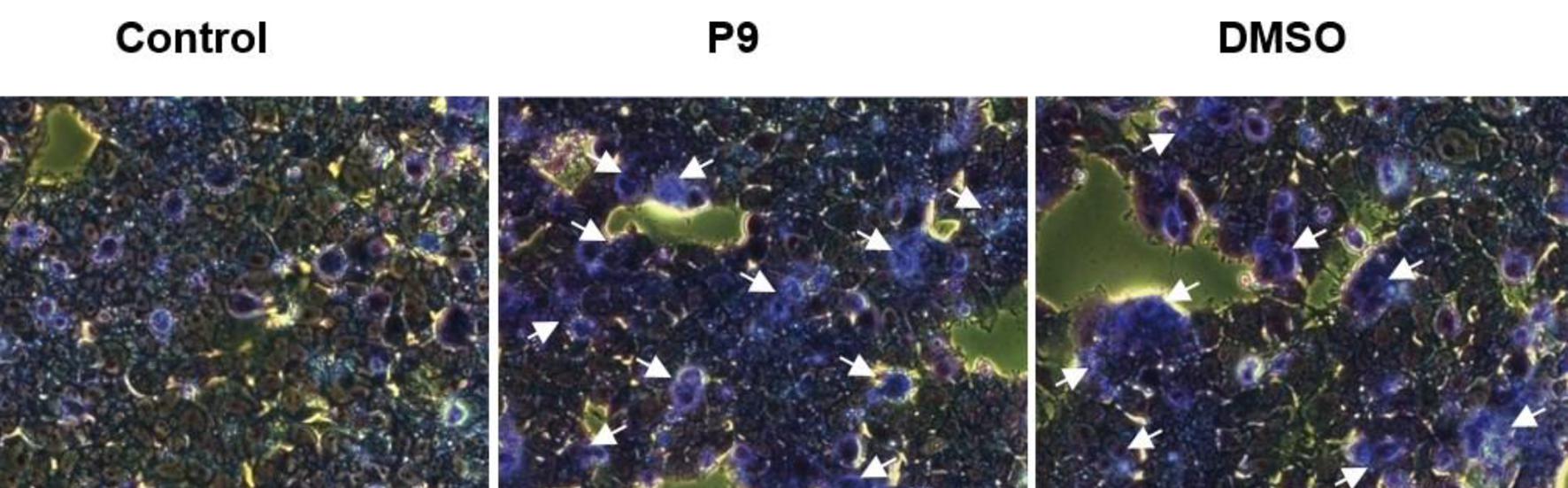


**Figure 6. Polykaryon formation by 293FT cells expressing HA protein after acidification at low pH.** P9 and 0.3% DMSO in DMEM (DMSO) were used to treat the cells before the cells were treated by low pH (5.0). The normal cells without HA expression were included as negative control (Control). Representative images were taken by microscope (original magnification 200×). The polykaryons are indicated by white arrows.


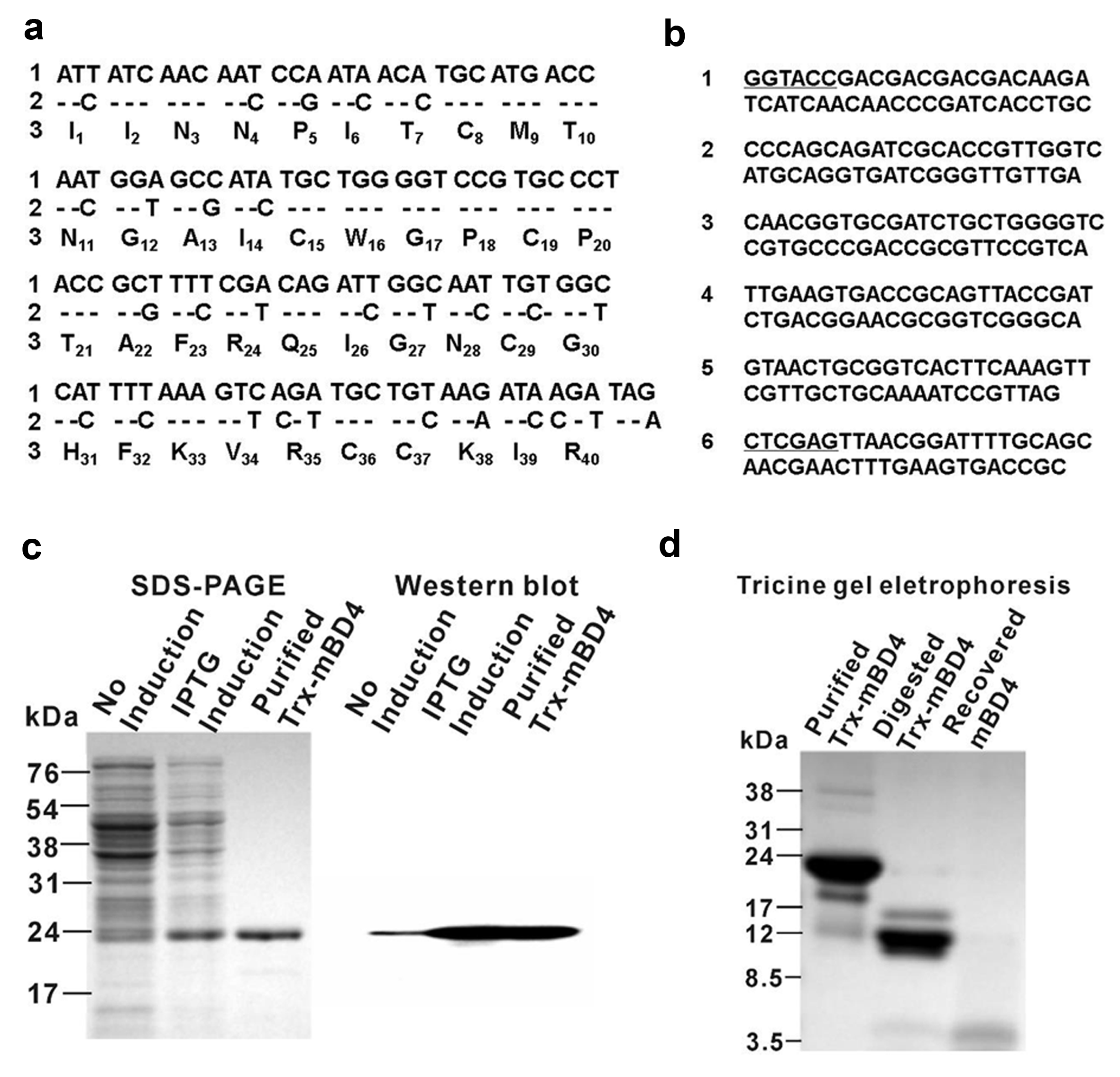


**Figure 7. Recombinant mBD4 was expressed by *E. coli* and purified by FPLC and enzyme digestion.** (**a**) The original codons of mBD4 were optimized to the *E. coli*-preferred codons by OPTIMIZER. (**b**) Based on the optimized sequence, six oligonucleotides were designed for PCR-based rmBD4 synthesis. The underlined nucleotides indicated recognition sites of KpnI and XhoI. (**c**) SDS-PAGE and Western blot analysis of the expression and purification of Trx-mBD4 fusion protein. (**d**) Tricine gel electrophoresis analysis of purified mBD4. Purified Trx-mBD4 was digested with enterokinase to release mBD4**.** mBD4 was recovered by cation-exchange chromatography. These protein samples were separated by 16% tricine gel electrophoresis. The final recovery rate of mBD4 was less than 5%.
